# Editorial: Children, Adolescents and Families With Severe Mental Illness: Toward a Comprehensive Early Identification of Risk

**DOI:** 10.3389/fpsyt.2021.812229

**Published:** 2021-12-21

**Authors:** Andrea Raballo, Frauke Schultze-Lutter, Marco Armando

**Affiliations:** ^1^Section of Psychiatry, Clinical Psychology and Rehabilitation, Department of Medicine, University of Perugia, Perugia, Italy; ^2^Center for Translational, Phenomenological and Developmental Psychopathology (CTPDP), Perugia University Hospital, Perugia, Italy; ^3^Department of Psychiatry and Psychotherapy, Medical Faculty, Heinrich-Heine-University, Düsseldorf, Germany; ^4^Department of Psychology, Faculty of Psychology, Airlangga University, Surabaya, Indonesia; ^5^University Hospital of Child and Adolescent Psychiatry and Psychotherapy, University of Bern, Bern, Switzerland; ^6^Service Universitaire de Psychiatrie de l'Enfant et de l'Adolescent, Centre Hospitalier Universitaire Vaudois (CHUV), Lausanne, Switzerland

**Keywords:** prevention, family, high risk, prognosis, severe mental disorder, suicide, developmental, psychopathology

Serious Mental Illnesses (SMI), such as depressive, bipolar, and psychotic disorders, often start in childhood and adolescence, are the leading cause of disability in young people and tend to cause life-long disability (1–4). SMI are commonly considered to originate from multiple, unfavorable and developmentally relevant gene-environment interactions; yet, the cause (or, more plausibly, the diachronic constellation of determinants and precipitating factors) of the SMI in an individual patient is usually unknown (5). Among epidemiological predictors, family disposition and early onset of mental problems are well-established predictors of SMI, in particular, when combined (6, 7). Furthermore, having a family member suffering from SMI profoundly affects family dynamics, e.g., by increasing expressed emotions, or by decreasing the patient's ability to support other family members while simultaneously increasing his or her need for their support (8, 9). On a converging line, it has been recently confirmed that, for example, preventive interventions targeting the offspring of parents with SMI have tangible prognostic impact both in terms of reduced incidence of mental illness in children and attenuation of internalizing symptoms (10, 11). However, within the framework of the early detection of psychosis, a positive family history of psychosis even in combination with a recent drop in functioning was an insufficient predictor of a conversion to first-episode psychosis by itself when compared to symptom-based risk criteria (12–14). Thus, contemporary clinical applied research on the early detection of SMI, even in its most developmentally-oriented branches, typically emphasizes an indicated approach based on subtle psychopathological antecedents (e.g., attenuated positive symptoms and basic symptoms [BS] as included in Clinical High Risk [CHR] criteria, anomalous subjective experiences, and schizotypal traits) (15) while the best strategy to incorporate evidence-based, transgenerational familial risk features still warrants more research (14, 16). Therefore, addressing the need of care and developing suitable early identification strategies for familial or genetic high-risk, and other young vulnerable groups is essential (11, 16–23).

In light of the above, this Research Topic aimed to disentangle some of the complexities in the field of children, adolescents and families with SMI, to advance knowledge on young people and families suffering from or being at risk of developing SMI and set the stage toward a comprehensive early identification of risk for SMI in children and adolescents (16, 20, 22, 23).

## Clinical High-Risk State for Psychosis and Familial Vulnerability: the Manifold Intersection of Risk

Nine papers in this Research Topic broadly addressed a clinical high-risk state (CHR) of psychosis in children and adolescents mainly by ultra-high risk (UHR) but also by basic symptom criteria (12, 13). Based on reports of high rates of personality disorders in adult UHR samples, Boldrini et al. examined personality traits in a 13–19-year-old Italian UHR sample (*n* = 58). Their results indicate that avoidant interpersonal strategies, impaired mentalization, and difficulties in emotional regulation might be important treatment targets to prevent both personality disorder and psychosis. Walger et al. examined the prevalence and the age effect of 14 criteria-relevant basic symptoms on clinical and functional patterns in a UHR sample (*N* = 261, age 15–40 yrs.). Results showed the high prevalence of basic symptoms (BS) in UHR and confirmed an age effect in BS and, thus, the earlier assumed link between presence of BS and brain maturation processes.

Poletti, Azzali, et al. investigated the prevalence of SMI in family members of distinct clinical subgroups of adolescents (*n* = 147; non-UHR vs. UHR vs. first-episode psychosis). Interestingly, results showed that more than 60% UHR had any broader family history of SMI, approximately a third of them by at least one first-degree relative. These results confirm the importance of within-family risk factors in UHR adolescents, suggesting the crucial need of their early detection. Consistently with the latter study, a clinical-conceptual perspective paper by Poletti, Gebhardt, et al. aimed to disentangle the complex intertwine of intergenerational risk factors that contribute to the risk of developing SMI in offspring, taking schizophrenia spectrum disorders as paradigmatic example.

In line with the UHR approach, Hartmann et al. offer initial insights on a broader, more agnostic approach to risk identification-to better capture the diffuse nature of emerging psychopathology, its developmental nuances and the multiplicity of potential exit syndromes other than psychosis (e.g., mania, severe depression, and personality disorder). Such extended approach, termed clinical high at-risk mental state [CHARMS (24)], might empower current opportunities for early risk inception, targeted early intervention and prevention strategies. On a complementary side, Kang-Yi et al. adopt an original angle to illuminate a widely neglected topic, that is the multitude of psychiatric diagnoses and related treatment that precede the recognition of schizophrenia in adolescents between 9 and 17 years (*n* = 1,459). The study confirmed earlier findings of multiple diagnoses and treatments initiated prior to, overall indicating a considerable need of care even in the prolonged help-seeking phase before the first and often delayed diagnosis of schizophrenia.

In a more basic research approach, Di Lorenzo et al. investigated the differences in auditory mismatch negativity (MMN) parameters in a 9–18-year-old sample of subjects with autism spectrum disorder (*n* = 37) with or without UHR by attenuated psychotic symptoms. The group with both conditions demonstrated a negative correlation between the severity of autistic symptoms and the MMN latency, although aberrations MMN amplitude and latency in the whole group were independent of concurrent attenuated psychotic symptoms. Büetiger et al. investigated the neural correlates of depersonalization and derealization through MRI in a mixed clinical sample of help-seeking individuals (CHR *n* = 97; clinical controls *n* = 91 and first-episode psychosis *n* = 29. Against the background of frequently depersonalization and derealization symptoms are frequent in CHR subjects, this study gives preliminary evidences that there may be divergent pathophysiological mechanisms leading to a final common pathway with similar psychopathological symptoms. Johnsen et al. conducted a systematic review on functional magnetic resonance imaging (fMRI) studies which examined task-related brain activity in young individual at familial high-risk for schizophrenia or bipolar disorder. Nineteen studies were selected. While the low number of studies and the substantial heterogeneity of employed methodological approaches impedes definite conclusions, all together these studies provide evidence for an altered brain processing of emotions in young individuals at familial high-risk for bipolar disorder.

## Depressive and Obsessive Features In Developmental Years

Four papers focus on depressiveness or obsessive-compulsivity in children and adolescents. Studying depression, sleep disorders and inflammatory factors in a 15–18-year-old mixed community-outpatient US sample (*n* = 92), Reddy et al. reported that one potential pathway between depressive symptoms and sleep disturbances in adolescents may be through an elevated tumor necrosis factor.

Two Chinese studies provided further support of an association between screen/internet use and depressiveness. In a large 10–15-year-old student sample (*n* = 14,500), Xu et al. showed that the consumption of fast food and sugar-sweetened beverages partially mediate the association between screen time and depressive symptoms by chain mediating effects. Furthermore, in a 11–15-year-old smaller student sample (*n* = 522), Chi et al. reported that a less positive youth development mediates the association between Internet addiction and depression, and that well-developed mindfulness can alleviate the negative effect of Internet addiction or a low level of psychological resources on depression.

Finally, Novara et al. present the validation of the Children's Yale-Brown Obsessive-Compulsive Scale (CY-BOCS) in a sample of children with OCD (*n* = 53) and Tourette Syndrome and TIC (*n* = 14). Their study indicates that a two-factor model of obsessions and compulsions represent appropriate measures for evaluating and monitoring the management of children with OCD.

## Empowering Mental Health Care Provision In Children and Adolescents

Five papers focussed on both general and specific aspects of mental health care provision in children and adolescents. Focussing on need for mental health care in unaccompanied refugee adolescents (*n* = 561) in Germany, Hanewald et al. reported relevant mental complaints in a 43.6% of the sample. The rates were affected by origin, age and sex, and substantiate the need for early detection of mental complaints and appropriate mental health care for at least every second unaccompanied refugee minor. In a Danish register study of children of psychiatric patients (*N* = 376) referred to child protection services, Ranning et al. found that only one third of children received support within 1 year after referrals from psychiatry, within an average of 3 months. Their results suggest that children of psychiatric patients represent an underserved population of children at risk for mental disorder. In a school setting study led in Spain, Romero-Ayuso et al. validated and tested (*n* = 103 teachers and *n* = 536 children) the potential usefulness of a questionnaire (EPYFEI-Escolar) aimed at determining academic needs and difficulties of children aged 3–11 yrs old. The results suggest the usefulness of this tool and shows that it can also be used to plan intervention programs in the school environment according to the needs of each child and school.

Capitalzing on an ongoing Norwegian innovative research project for child and adolescent mental health disorders, i.e., the Individualized Digital DEcision Assist System, Røst et al. present a model to adapt and harness technological advancements such as computerized Clinical Decision Support Systems, to support practitioners in providing evidence-based care in child and adolescent mental health services.

Finally, given the increased risk of mental disorders in children with a positive family history of SMI, Wiegand-Grefe et al. discuss the architecture of a family-based intervention program for children of mentally ill parents (CHIMPs) whose overall scheme consists of a two-group randomized controlled multicenter trial of soft-psychosocial intervention involving families with mentally ill parents and their children aged 3–19 years.

## Intergenerational Risk in Other Psychopathological Spectra: Autism, 22q11.2 Deletion Syndrome, and Non-Suicidal Self-Injury

Three papers addressed intergenerational risk features in relevant, adjacent areas of psychopathology, such as autism-spectrum disorder (ASD), psychopathological expressions of 22q11.2 deletion syndrome, and non-suicidal self-injury (NSSI). Coelho and Conceição  investigated whether parental perceptions of the disorder could contribute to a better outcome prediction of children with ASD (*n* = 55). Together, the findings of this study suggest that parents' concerns should be taken into account when planning a therapeutic intervention for ASD. Sandini et al. focused on the 22q11.2 deletion syndrome (aka DiGeorge syndrome and velocardiofacial syndrome), which is characterized by an extended and highly variable psychiatric phenotype across subjects (including schizophrenia, anxiety disorders, mood disorders, and Attention Deficit Hyperactivity Disorder) to look for bi-directional interactions of parental anxious-depressive features and offspring psychopathology. The results confirm an intergenerational association between high levels of parental anxiety and depression and increased psychopathology in offspring for both internalizing and externalizing symptoms. Fu et al. focus on the wide domain of non-suicidal self-injury through a qualitative research design and specifically explore parents' attitudes toward and perceptions of adolescents who have engaged in such behaviors. The study highlights important neglected aspects of the broader non-suicidal self-injury behavior impact on the family, in particular that parents suffer great emotional stress and often lack the knowledge about non-suicidal self-injury and its treatment.

## Conclusion

Overall, we believe that this Research Topic on “Children, Adolescents and Families with Severe Mental Illness: Toward a Comprehensive Early Identification of Risk” that will be continued as a Community Series provides a comprehensive rationale for rethinking a new wave of broad, family-sensitive approaches to better understand the determinants of SMI in children and adolescents and preventing mental ill-health.

## Author Contributions

AR, FS-L, and MA contributed substantially to the development of this special issue in the conceptual planning as well as in the reviewing and editing of the included papers. Similarly, they jointly contributed to the preparation and review of the editorial manuscript. All authors contributed to the article and approved the submitted version.

## Conflict of Interest

The authors declare that the research was conducted in the absence of any commercial or financial relationships that could be construed as a potential conflict of interest.

## Publisher's Note

All claims expressed in this article are solely those of the authors and do not necessarily represent those of their affiliated organizations, or those of the publisher, the editors and the reviewers. Any product that may be evaluated in this article, or claim that may be made by its manufacturer, is not guaranteed or endorsed by the publisher.
